# Dioecious plants are more precocious than cosexual plants: A comparative study of relative sizes at the onset of sexual reproduction in woody species

**DOI:** 10.1002/ece3.3117

**Published:** 2017-06-15

**Authors:** Itsuki Ohya, Satoshi Nanami, Akira Itoh

**Affiliations:** ^1^ Graduate School of Science Osaka City University Osaka Japan

**Keywords:** angiosperm, cosexuality, dioecy, onset of sexual reproduction, sex expression, woody plant

## Abstract

The reproductive capacities of dioecious plant species may be limited by severe pollen limitation and narrow seed shadows for the two reasons. First, they are unable to self‐pollinate, and seed production occurs only with pollinator movement from males to females. Second, only 50% of the individuals in populations contribute to seed production. Despite these handicaps, dioecious plants maintain their populations in plant communities with cooccurring cosexual plants, and no substantial difference in population growth rates has been found between dioecious and cosexual plants. Hence, dioecious plants are thought to mitigate these disadvantages by adopting ecological traits, such as insect pollination, animal‐dispersed fleshy fruits, and precocious flowering. We studied the relationship between flowering and plant size in 30 woody species with different sex expressions, leaf habits, fruit types, and maximum plant sizes. The study site was located in an evergreen broad‐leaved forest on the island of Honshu, Japan. A phylogenetic linear regression model showed that dioecious species tended to mature at smaller sizes than did cosexual taxa. At the population level, given equal plant densities and reproductive efforts, the precocity of dioecious plants could serve as one of the factors that mitigate the limitations of pollen and seed‐shadow handicaps by increasing the density of reproductive individuals in the population. At the individual level, smaller size of onset of flowering may play a role in enhancing reproductive success over a lifetime by increasing reproductive opportunities. We discussed the possible effect of the relationship between precocity and some ecological traits of dioecious plants, such as small flowers pollinated by unspecialized insects, fleshy fruit dispersed by animals, and their preferential occurrence in the tropics and in island habitats. The universality of precocity among dioecious plants should be investigated in diverse plant communities. Such studies will increase our understanding of the evolution of plant breeding systems.

## 1 INTRODUCTION

Populations of dioecious plant species may grow more slowly than those of cooccurring cosexual taxa for two reasons. Firstly, dioecious plants are likely to suffer from pollination limitation because they are unable to self‐pollinate and require another individual to mate with for seed production (Schlessman, Vary, Munzinger, & Lowry, 2014; Wilson & Harder, 2003; Xia et al., 2013; __ster & Eriksson, 2007). Although self‐incompatible plants also require another individual to mate with, all interplant pollinator movement potentially contributes to seed production. For dioecious plants, however, the direction of pollinator movement is an important factor, because only the movements from males to females enable seed production (Vamosi, Vamosi, & Barrett, 2006). Several field studies have suggested that pollen limitation in dioecious plants is the main factor limiting seed set. The fruits set of flowers and seed production per fruit were reduced when the density of reproductive males was low (Ashman & Diefenderfer, 2001; Xia et al., 2013; __ster & Eriksson, 2007). Furthermore, the fruit set of dioecious trees increased in females when males occurred in nearby sites (House, 1992; de Jong, Batenburg, & Klinkhamer, 2005; Otero‐Arnaiz & Oyama, 2001). In a gynodioecious shrub, *Daphne laureola*, the pollination deficit was greater for females than for hermaphrodites (Medrano, Alonso, & Herrera, 2005). Secondly, the ability to colonize uninhabited sites is relatively limited in dioecious species because only 50% of the individuals in a population contribute to seed production (Heilbuth, Ilves, & Otto, 2001; Vamosi, Zhang, & Wilson, 2007). The seed‐shadow handicap has been demonstrated in field studies. In dioecious tree species, seed dispersal was limited to the vicinity of female plants, resulting in a strongly clumped distribution of seedlings (Montesinos, Verdú, & García‐Fayos, 2007; Nanami, Kawaguchi, & Yamakura, 1999). These disadvantages would adversely affect the colonizing ability of dioecious plants (Baker, 1955). Wilson and Harder (2003) examined competition for space between dioecious and cosexual species using mathematical models. They showed that separation of the sexes increased the variance in the fertilization probability and seed production among plants, and the reproductive uncertainty reduced the mean recruitment of the dioecious species. On the other hand, Bruijning et al. (2017) suggested that the cost of having males was smaller than generally expected and dioecious species maintain similar population growth rates to those of cosexuals through higher seed production of dioecious females than that of cosexuals. Analyses based on field studies as Bruijning et al. (2017) are still scarce at present, practical field data would be needed.

Despite the potential disadvantages, 43% of angiosperm families have dioecious members (Renner, 2014; Renner & Ricklefs, 1995), and the evolution of dioecy has occurred independently many times across a diverse range of taxonomic groups (Barrett, 2013; Renner, 2014). Furthermore, dioecious plant species occur in global plant communities, although tropical or island flora has higher incidences of dioecious species compared to the global proportion of 6% (Bawa, 1980; Ibarra‐Manriquez & Oyama, 1992; Queenborough et al., 2009; Sakai, Wagner, Ferguson, & Herbst, 1995a; Vary et al., 2011). The taxonomical and geographical ubiquity of dioecious species suggests that they compensate for their reproductive disadvantages through ecological traits that allow them to coexist with cosexual competitors.

The ecological traits associated with dioecy have been explored and debated (Pickup & Barrett, 2012; Renner & Ricklefs, 1995; Vamosi, Mazer, & Cornejo, 2008; Vamosi, Otto, & Barrett, 2003; Vary et al., 2011). For example, the correlation between insect pollination and dioecy is strong, especially in wet evergreen forest (Bawa, 1980). Beach (1981) discussed that insect pollination favours dioecious plants because it promotes directional pollinator movement from more‐flowered individuals (males) to less‐flowered individuals (females). Additionally, many dioecious species have fleshy fruit that are dispersed mainly by birds that transport seeds over long distances (Bawa, 1980; Renner & Ricklefs, 1995; Sakai & Weller, 1999; Vamosi et al., 2003; Vary et al., 2011). This form of zoochory would reduce the seed‐shadow handicap of dioecious plants (Heilbuth et al., 2001; Vamosi et al., 2007). Dioecy is significantly associated with long‐lived, woody growth forms (Renner & Ricklefs, 1995; Sakai, Wagner, Ferguson, & Herbst, 1995b; Vamosi et al., 2003). Dioecious woody species are probably able to survive a season of reproductive failure (Renner, 2014); furthermore, the probability of finding a reproductive mate during the course of a long life span is likely to be high in dioecious taxa (Pannell & Barrett, 1998).

The problem is the compensatory mechanisms of various ecological traits of dioecious plants, including the foregoing, have not been documented in field studies. To resolve this problem, Bruijning et al. (2017) first analyzed field demographic data covering the entire life cycles of trees in tropical moist forests in Panama. They showed that the disadvantages of dioecy are largely negated in long‐lived plants mainly by the larger number of seeds produced by dioecious females compared with cosexual species. Although field data analyses, such as those performed by Bruijning et al. (2017), are critical for understanding the mechanism of the coexistence of dioecious and cosexual plants, a few specific mechanisms of compensation remain unstudied.

Among the poorly studied ecological traits, precocity is considered a likely candidate to serve as the mechanism that compensates for the reproductive disadvantages of dioecy (Queenborough et al., 2009). The ontogenetic stage at which an organism begins to reproduce has a large influence on population growth rates (Gadgil & Bossert, 1970), and the age and/or size in timing of first flowering has been an important topic in studies on life history of plants (Lacey, 1986; Wenk & Falster, 2015). Nevertheless, comparative studies of the onset of sexual maturity among plant species are not so many to date (but see Thomas, 1996; Davies & Ashton, 1999; Wright et al., 2005; Bruijning et al., 2017). Using an entire life‐cycle approach to evaluate the fitness consequences of dioecy and cosexuality, Bruijning et al. (2017) found little difference in the threshold size of flowering between dioecious and cosexual trees in tropical moist forests in Panama. However, Bruijning et al. (2017) are the only study in which the correlation between sex expression and size at the onset of the maturity of plants has been examined, and field evidence is still scarce. Therefore, the onset of sexual maturity in dioecious and cosexual plants should be investigated in diverse plant communities.

Here, we compared the precocity of flowering in 11 dioecious and 19 cosexual woody species in a temperate evergreen broad‐leaved forest. We evaluated the precocity of flowering using plant size, and not age, for the following reasons. First, age and size are not accurate proxies for each other in woody plants. The correlation between age and size of woody plants is often significant but weak (Ågren & Zackrisson, 1990; Bradley, 1997; Collet, Fournier, Ningre, Hounzandji, & Constant, 2011; Rebertus, Burns, & Veblen, 1991), because environmental conditions affect their vegetative growth rates (Nanami, Kawaguchi, & Yamakura, 2005, 2011; Santos‐del‐Blanco, Bonser, Valladares, & Chambel, 2013). Consequently, plants of the same age may be of different sizes (Martínez‐Ramos & Alvarez‐Buylla, 1998), and large plants are not necessarily old (Korning & Balslev, 1994). Second, size is a good predictor of sexual maturity in plants (Augspurger, 1985; Itoh et al., 2003; Lacey, 1986; Nanami et al., 2005; Otárola, Sazima, & Solferini, 2013; Wesselingh, Klinkhamer, de Jong, & Boorman, 1997), and the onset of reproduction is often related to size rather than age, especially for long‐lived perennials (Barot, Mitja, Miranda, Meija, & Grimaldi, 2005; de Jong & Klinkhamer, 2005). Indeed, previous studies have estimated the onset of flowering of woody plants based on plant size (Barot et al., 2005; Bruijning et al., 2017; Davies & Ashton, 1999; Santos‐del‐Blanco et al., 2013; Thomas, 1996; Wright et al., 2005).

We examined the relationships of plant size at the onset of flowering with (1) sex expression (dioecious vs. cosexual); (2) ecological traits examined in previous studies, i.e., leaf habit (deciduous vs. evergreen), fruit type (fleshy vs. dry), and growth form (tree vs. shrub); and (3) phylogenetic constraints.

## 2 MATERIALS AND METHODS

### 2.1 Study site

Field surveys were performed in the Kasugayama Forest Reserve, Nara City, Japan (34°41′N, 135°51′E: elevation: 498 m on the highest peak). The area was covered by a well‐preserved climax forest dominated by evergreen oaks, (e.g., *Castanopsis cuspidata*) accompanied by other evergreen broad‐leaved trees (e.g., *Neolitsea aciculata* and *Symplocos prunifolia*) and deciduous broad‐leaved trees (e.g., *Acer rufinerve* and *Cornus macrophylla*) (Shimoda, Kimura, Kanzaki, & Yoda, 1994). Pioneer species, such as *Aralia elata*,* Mallotus japonicus,* and *Zanthoxylum ailanthoides*, occurred occasionally (Naka & Yoneda, 1984). The reserve covered an area of *ca*. 300 ha. As a divine forest belonging to the Kasuga Shinto Shrine, the reserve has been protected from logging for >1,000 year. The mean annual temperature and mean annual precipitation during 1981–2010 were 14.9°C and 1,316 mm/year, respectively, at the Nara Meteorological Station (elevation: 104 m), located *ca*. 2 km northwest of the study site.

### 2.2 Materials

We investigated 30 tree and shrub species belonging to 26 genera in 18 families (Table 1). These species varied in their ecological traits, i.e., sex expression, leaf habit, fruit type, and maximum plant size. Other than *Camellia japonica*, which is bird pollinated (Kunitake, Hasegawa, Miyashita, & Higuchi, 2004), all of the species are insect pollinated.

**Table 1:** Characteristics of the studied species. *S*_0.5_, 95th DBH and *N* indicate the relative plant size at which 50% of the individuals bore flowers, the 95th percentile of stem diameter at breast height and number of observed individuals, respectively

Species	Family	*S*_0.5_	Sex expression	Leaf habit	Fruit tipe	95th DBH (cm)	*N*
*Magnolia obovata*	Magnoliaceae	0.358	Cosexual	Deciduous	Fleshy	56.1	41
*Magnolia salicifolia*	Magnoliaceae	0.377	Cosexual	Deciduous	Fleshy	30.3	66
*Neolitsea aciculata*	Lauraceae	0.244	Dioecious	Evergreen	Fleshy	23.4	131
*Neolitsea sericea*	Lauraceae	0.113	Dioecious	Evergreen	Fleshy	26.0	58
*Litsea coreana*	Lauraceae	0.284	Dioecious	Evergreen	Fleshy	71.0	21
*Machilus japonica*	Lauraceae	0.372	Cosexual	Evergreen	Fleshy	38.1	45
*Cinnamomum yabunikkei*	Lauraceae	0.081	Cosexual	Evergreen	Fleshy	30.7	47
*Symplocos prunifolia*	Symplocaceae	0.668	Cosexual	Evergreen	Fleshy	20.3	34
*Styrax japonica*	Styracaceae	0.186	Cosexual	Deciduous	Dry	25.3	58
*Pieris japonica*	Ericaceae	0.537	Cosexual	Evergreen	Dry	16.3	73
*Lyonia ovalifolia*	Ericaceae	0.397	Cosexual	Deciduous	Dry	10.3	73
*Clethra barbinervis*	Clethraceae	0.393	Cosexual	Deciduous	Dry	18.2	71
*Camellia japonica*	Theaceae	0.214	Cosexual	Evergreen	Dry	33.1	40
*Eurya japonica*	Pentaphylacaceae	0.210	Dioecious	Evergreen	Fleshy	7.9	76
*Gamblea innovans*	Araliaceae	0.216	Dioecious	Deciduous	Fleshy	30.3	52
*Chengiopanax sciadophylloides*	Araliaceae	0.280	Cosexual	Deciduous	Fleshy	29.6	58
*Aralia elata*	Araliaceae	0.150	Cosexual	Deciduous	Fleshy	16.2	38
*Ilex pedunculosa*	Aquifoliaceae	0.140	Dioecious	Evergreen	Fleshy	28.3	81
*Osmanthus heterophyllus*	Oleaceae	0.101	Dioecious	Evergreen	Fleshy	23.7	82
*Ligustrum japonicum*	Oleaceae	0.277	Cosexual	Evergreen	Fleshy	12.8	31
*Cornus macrophylla*	Cornaceae	0.388	Cosexual	Deciduous	Fleshy	40.4	47
*Castanopsis cuspidata*	Fagaceae	0.358	Cosexual	Evergreen	Dry	54.2	40
*Photinia glabra*	Rosaceae	0.220	Cosexual	Evergreen	Fleshy	40.7	40
*Laurocerasus spinulosa*	Rosaceae	0.284	Cosexual	Evergreen	Fleshy	57.6	22
*Mallotus japonicus*	Euphorbiaceae	0.062	Dioecious	Deciduous	Dry	34.8	91
*Zanthoxylum piperitum*	Rutaceae	0.136	Dioecious	Deciduous	Dry	6.4	41
*Zanthoxylum ailanthoides*	Rutaceae	0.221	Dioecious	Deciduous	Dry	46.9	70
*Acer palmatum*	Sapindaceae	0.327	Cosexual	Deciduous	Dry	38.4	57
*Acer rufinerve*	Sapindaceae	0.087	Dioecious	Deciduous	Dry	35.3	155
*Illicium anisatum*	Schisandraceae	0.422	Cosexual	Evergreen	Dry	16.6	50

During the flowering season of each species, we searched for the target taxa along small trails in the forest. Following the procedures performed by Thomas (1996), we collected data on *A*. *elata*,* M*. *japonicus*, and *Z*. *ailanthoides* in a nearby disturbed roadside area, as these pioneer species were rare within the primary forest. When individuals of a target species were located, we observed the flowers through binoculars. We also recorded the genders of individuals of dioecious species. We measured stem diameters at breast height (130 cm above ground level, DBH). We measured the stem diameters of *Z*. *piperitum*, a small shrub, at ground level. The survey was continued until adequate numbers of individuals (≥20) in various stem‐size categories had been examined. The sample size of each species (Table 1) depended on its abundance in the forest. The field surveys of 28 of the 30 species were conducted during March–November in 2016. Data for *L*. *coreana* and *N*. *aciculata* were collected in 1992. Measurements on *N*. *aciculata* were limited to medium‐sized individuals. We therefore collected additional information on small and large individuals in 2016.

### 2.3 Statistical analyses

**Figure 1:**
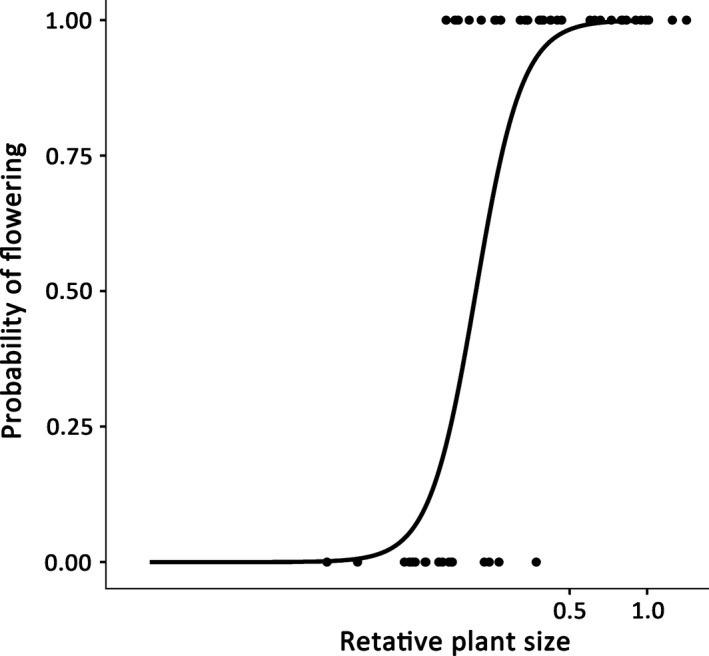
Example of a logistic regression curve representing probability of flowering (*P*) as a function of the relative plant sizes (the stem diameter divided by the 95th percentile of measured stem diameters) of *Gamblea innovans* (Araliaceae) (*N *= 52) in an evergreen broad‐leaved forest, Japan. Fitted curve: *P *= exp(7.378 + 4.812ln*S*)/(1 + exp(7.378 + 4.812ln*S*). The relative plant size at which 50% of the individuals bore flowers (*S*_0.5_) is calculated as exp(−7.378/4.812) = 0.216

We used the logistic regression function to describe the relationship between a binary variable (flowering vs. nonflowering) and a continuous variable (relative plant size) (Figure 1). This regression has been used to describe the size dependence of the onset of reproduction in both plants (Thomas, 1996) and animals (Giménez & Penchaszadeh, 2003). The relative plant size of an individual was calculated by dividing the stem diameter by the estimated maximum stem diameter for the species. In this study, the estimated maximum stem diameter was defined as the 95th percentile of measured stem diameters for each species. We standardized our observed values using the 95th percentile instead of the observed maximum value to increase the robustness of the analysis and reduce sensitivity to outliers. We used the following regression equation for this analysis:

$$P=\frac{e^{a+b\text{lnS}}}{(1+e^{a+b\text{lnS})}},$$

where *P* is the probability of flowering, *S* is the relative plant size of an individual, *e* is the base of the natural logarithm, and *a* and *b* are constants. The regression was calculated using R ver. 3.2.3 software (R Development Core Team 2015, https://cran.r-project.org/bin/windows/base/old/3.2.3/). Following the procedures of Giménez and Penchaszadeh (2003), we determined the onset of flowering at the population level as the relative plant size at which 50% of the individuals bore flowers (*S*_0.5_). After substituting 0.5 for *P*, we obtained the following transformation of the equation: *S*_0.5_ = exp(−*a*/*b*).

**Figure 2:**
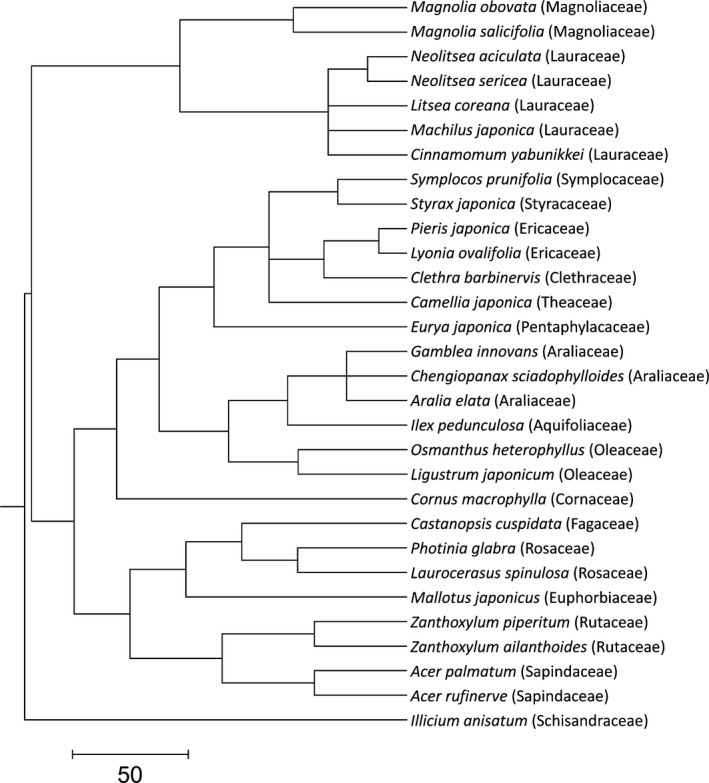
Phylogenetic relationships of 30 woody species in an evergreen broad‐leaved forest, Japan

The phylogenetic relationships among the 30 species (Figure 2) were determined using Phylomatic ver. 3, based on an APG‐III‐derived supertree (R20120829) (Webb & Donoghue, 2005). The branch lengths were adjusted using the *bldj* algorithm in the Phylocom 4.2 software (Webb, Ackerly, & Kembel, 2008) using an ages file, agescl3 (Gastauer & Meira‐Neto, 2013), and a pairwise phylogenetic distance matrix was generated.

The phylogenetic signal of *S*_0.5_ was evaluated by Blomberg's *K* (Blomberg, Garland, & Ives, 2003) and Pagel's λ (Pagel, 1999) using the phylosig function of the R *phylotools* package (Zhang, Mi, & Pei, 2012). Statistical significance was tested with 999 permutations of species for *K* and the likelihood ratio test for λ. Multichotomies were resolved using the multi2di function of the R *ape* package (Paradis, Claude, & Strimmer, 2004).

Factors affecting *S*_0.5_ were tested with a phylogenetic linear regression using the above‐mentioned phylogeny. The response variable was *S*_0.5_ for each species, and the explanatory variables were (1) sex expression (dioecious vs. cosexual), (2) leaf habit (deciduous vs. evergreen), (3) fruit type (fleshy vs. dry), and (4) the 95th percentile of stem diameter. The 95th percentile of stem diameters represents the growth form (tree vs. shrub) of each species. We calculated all possible models and selected the best‐fitting model based on Akaike's information criterion (AIC). The regression was calculated with the R *phylolm* package, version 2.5 (Ho & Ané, 2014) using Pagel's λ model.

## 3 RESULTS

The focal group of 30 species comprised 11 dioecious and 19 cosexual taxa (Table 1). *S*_0.5_ was a nonsignificant phylogenetic signal (*K *= 0.407, *P *= .346; λ = 0.101, *P *= .837), indicating that closely related species did not always flower at similar relative sizes. The four focal families in the study included species with different sex expressions (Table 1). Members of the Lauraceae comprised three dioecious and two cosexual species. The Araliaceae included one dioecious and two cosexual species. The Oleaceae and Sapindaceae each had one dioecious and one cosexual species. Within individual families, the dioecious species had smaller *S*_0.5_ values compared with cosexual ones, except for Araliaceae.

**Figure 3:**
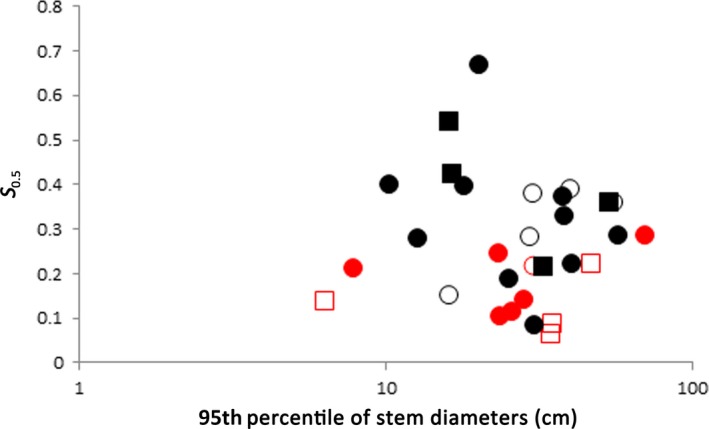
Scatter plot showing the relationship between the 95th percentile of measured stem diameters and the relative plant size at which 50% of the population flowered (*S*_0.5_). Red and black symbols indicate dioecious and cosexual species, respectively. Solid and open symbols represent evergreen and deciduous species, respectively. Squares and circles indicate dry and fleshy fruits, respectively

Dioecious species tended to mature at a smaller relative size than cosexual species (Figure 3). The average ± standard deviation of relative size at onset of maturity were 0.165 ± 0.073 and 0.331 ± 0.136 for dioecious and cosexual species, respectively (Table 1).

The phylogenetic linear regression model identified significantly different *S*_0.5_ values in dioecious than in cosexual species (Table 2). No significant effect of other variables on *S*_0.5_ was detected among the 30 species.

**Table 2:** The effects of four ecological traits on the relative plant size at which 50% individuals in a population flower (*S*_0.5_). Effects were estimated using a phylogenetic linear regression model. (a) The full model with all available traits and (b) the best‐fit model selected based on Akaike's information criterion (AIC)

	Estimated coefficient	Standard error	*t*‐Value	*P*‐value
(a)				
(Intercept)	3.26 × 10^–1^	5.89 × 10^–2^	5.53	9.45 × 10^–6^
Sex expression	−1.66 × 10^–1^	4.65 × 10^–2^	−3.57	1.49 × 10^–3^
Leaf habit	3.55 × 10^–2^	4.75 × 10^–2^	0.747	0.462
Fruit type	−1.56 × 10^–2^	4.89 × 10^–2^	−0.320	0.752
Max. DBH	−1.55 × 10^–4^	1.48 × 10^–3^	−0.105	0.917
(b)				
(Intercept)	0.331	2.69 × 10^–2^	12.3	8.02 × 10^–13^
Sex expression	−0.166	4.43 × 10^–2^	−3.74	8.34 × 10^–4^

(a) Lambda = 5.55 × 10^–9^, AIC = −32.3.

(b) Lambda = 7.62 × 10^–9^, AIC = −37.6.

## 4 DISCUSSION

Dioecious plants are considered to have two reproductive disadvantages compared to cosexual plants, i.e., stronger pollen limitation and seed‐shadow handicap. Queenborough et al. (2009) suggested precocious reproduction as a mechanism of compensation for the disadvantages. Although the reproductive size thresholds of long‐lived plants has been studied in several studies (Barot et al., 2005; Davies & Ashton, 1999; Santos‐del‐Blanco et al., 2013; Thomas, 1996; Wright et al., 2005), Bruijning et al. (2017) are the only one to examine the association between the sex expression of plants and their size at the onset of flowering. They found little difference in the threshold size of flowering between dioecious and cosexual trees in tropical moist forests in Panama. In comparison, dioecious plants were more precocious than cosexuals in our study (Figure 3 and Table 2). To confirm the universality of precocity in dioecious plants, we reviewed the studies conducted by Thomas (1996) and Wright et al. (2005) that provide comparative data on the onset of reproduction between dioecious and cosexual plants. Thomas (1996) provided data on 28 dioecious species and nine cosexual species. The averages ± standard deviations of the relative size at the onset of maturity were 0.408 ± 0.147 and 0.588 ± 0.294 for dioecious and cosexual species, respectively. Wright et al. (2005) included five dioecious species and seven cosexual species, with an averages ± standard deviations of the relative size at the onset of maturity of 0.578 ± 0.116 and 0.627 ± 0.147 for dioecious and cosexual species, respectively. These data support our hypothesis.

The size at which an organism begins to reproduce has a large influence on population growth rates (Gadgil & Bossert, 1970) and the fitness of individuals (Wenk & Falster, 2015). The precocity of dioecious plants should alleviate their reproductive disadvantages at the population level. Firstly, precocity increases the density of reproductive male plants, thereby reducing the negative effect of pollen limitation. Secondly, precocity increases the density of flowering female plants, thereby extending the seed shadow. Queenborough et al. (2009) predicted that the increased population densities of dioecious species compensate for the reproductive disadvantages of this form of sex expression, but they found no difference in the abundance between dioecious species and their most closely related cosexual species in western Amazonia. However, total density is not so relevant in this context. The density of reproductive individuals may be much more important for testing the predictions of Queenborough et al. (2009). As we showed in this study, the precocity of dioecious plants increases the density of individuals participating in reproduction.

At the individual level, a theoretical study indicated that dioecious plants must have more than twice the fecundity of cosexuals (Charlesworth & Charlesworth, 1978) to offset the loss of one sexual function. However, empirical data suggested that the fecundity of dioecious plants at a flowering event was lower than the required value (Asikainen & Mutikainen, 2003; Ibarra‐Manriquez & Oyama, 1992; Medrano et al., 2005). Smaller size at the onset of flowering may enhance reproductive success over a lifetime by increasing reproductive opportunities. The disadvantages of dioecious plants would be fully compensated for by a combination of several ecological traits, including precociousness, larger numbers (but less than twice compared to cosexuals) of flowers and/or fruits (e.g., Ibarra‐Manriquez & Oyama, 1992), and preferential foraging of animal seed dispersers on dioecious females (Vamosi et al., 2007), but not by any one ecological trait. A comprehensive estimate of the cumulative effects of multiple traits is necessary to explain how dioecious plants conquer their reproductive disadvantages (Bruijning et al., 2017).

The precocity of dioecious plants is possible to associate with some reported ecological traits. Dioecious plants tend to have small flowers pollinated by unspecialized, opportunistic insects and fleshy fruit dispersed by animals (Bawa, 1980; Ibarra‐Manriquez & Oyama, 1992). Thompson (2001) reported that the number of visits by opportunistic pollinators was positively related to the number of open flowers in a patch of a shrub species, but such a positive relationship between floral display and visitation rate was not true for specialist pollinators. The precocity of dioecious plants may facilitate their reproduction by attracting generalist pollinators via increased reproductive plant density. The effect of the high density of reproductive plants also applies to seed dispersal mutualism between plants and animals. Avian seed dispersers tended to visit stands with large crops (Christensen & Whitham, 1991). In populations of dioecious plants, an attractive display for seed dispersers may be achieved in part by their precocity which increases the density of fruiting individuals in a population.

The precocity of dioecious plants might relate to the geographical variation in their distributions. Globally, dioecious taxa account for only 6% of angiosperm species (Renner, 2014; Renner & Ricklefs, 1995), but the proportions are much higher in tropical (Bawa, Perry, & Beach, 1985; Ibarra‐Manriquez & Oyama, 1992; Queenborough et al., 2009; Vary et al., 2011) and island habitats (Sakai & Weller, 1999; Sakai et al., 1995a). Tropical forests have an extreme richness of tree species and low density of each species compared to temperate forests (Ashton, 1988; Janzen, 1970). The low density of tropical tree species is likely to reduce their reproductive output through pollen limitation (Murawski & Hamrick, 1991). The effect of precocity of dioecious plants that reduces the difference with cosexuals may more effective in tropics where plant density is low for both of dioecious and cosexual species.

On islands, dioecious plants may survive better than cosexuals due to outbreeding, which increases genetic variation, and by decreasing inbreeding depression (Bawa, 1980). In this study, we add precocity as another possible factor underlying the prevalence of dioecious plants on islands. Island populations are considered to have higher risks of extinction than mainland populations partly due to small size of populations (Frankham, 1998; Rosenzweig & Clark, 1994). Precocious reproduction may help dioecious species to avoid extinction by increasing population growth rates. Furthermore, a smaller threshold size of reproduction would mean, to some extent, that they reproduce earlier in their lives. Plant species that reproduce early would succeed in colonization of new habitats (Colautti & Barrett, 2013; Dangremond & Feller, 2016) because individuals produce offspring before their accidental death. The precocity of dioecious plants might enable them to colonize islands, particularly after migration by a few founders. The universality of precocity in dioecious plants should be investigated in diverse plant communities to explain the compensation mechanism against reproductive disadvantages and geographical variation in the abundance of dioecious species.

## CONFLICT OF INTEREST

None declared.
